# *Degree of stemness* predicts micro-environmental response and clinical outcomes of diffuse large B-cell lymphoma and identifies a potential targeted therapy

**DOI:** 10.3389/fimmu.2022.1012242

**Published:** 2022-11-08

**Authors:** Fang Hu, Huan Li, Lei Li, Robert Peter Gale, Yuanbin Song, Shuiqin Chen, Yang Liang

**Affiliations:** ^1^ Sate Key Laboratory of Oncology in South China, Sun Yat-sen University Cancer Center, Collaborative Innovation Center for Cancer Medicine, Guangzhou, China; ^2^ Department of Hematologic Oncology, Sun Yat-sen University Cancer Center, Guangzhou, China; ^3^ Department of ICU, State Key Laboratory of Oncology in South China, Collaborative Innovation Center for Cancer Medicine, Sun Yat-sen University Cancer Center, Guangzhou, China; ^4^ Guangdong Provincial Key Laboratory of Malignant Tumor Epigenetics and Gene Regulation, Guangdong-Hong Kong Joint Laboratory for RNA Medicine, Medical Research Center, Sun Yat-sen Memorial Hospital, Sun Yat-sen University, Guangzhou, China; ^5^ Haematology Research Centre, Department of Immunology and Inflammation, Haematology Research Centre, Imperial College London, London, United Kingdom

**Keywords:** diffuse large B-cell lymphoma, stem cells, targeted therapy, single-sample gene set enrichment analysis, weighted gene co-expression network analysis, dequalinium chloride

## Abstract

Some cells within a diffuse large B-cell lymphoma (DLBCL) have the genotype of a stem cell, the proportion of which is termed *degree of stemness*. We interrogated correlations between the *degree of stemness* with immune and stromal cell scores and clinical outcomes in persons with DLBCL. We evaluated gene expression data on 1,398 subjects from Gene Expression Omnibus to calculate the *degree of stemness*. Subjects were classified into low- and high-stemness cohorts based on restricted cubic spline plots. Weighted gene co-expression network analysis (WGCNA) was used to screen for stemness-related genes. Immune and stromal scores correlated with the *degree of stemness* (both P < 0.001). A high *degree of stemness* correlated with a shorter progression-free survival (PFS; Hazard Ratio [HR; 95% Confidence Interval [CI] =1.90 (1.37, 2.64; *P* < 0.001) and a shorter survival (HR = 2.29 (1.53, 3.44; P < 0.001). *CDC7* expression correlated with the *degree of stemness*, and CDC7-inhibitors significantly increased apoptosis (*P* < 0.01), the proportion of cells in G_1_ phase (*P* < 0.01), and inhibited lymphoma growth in a mice xenograft model (*P* = 0.04). Our data indicate correlations between the *degree of stemness*, immune and stromal scores, PFS, and survival. These data will improve the prediction of therapy outcomes in DLBCL and suggest potential new therapies.

## Introduction

Most, if not all, cancers arise from a transformed stem cell or a more differentiated cell, which has acquired a stem cell genotype with self-renewal capacity (1, 2). This is especially so for hematopoietic cancers, including lymphomas (3–5). The proportion of cells within a cancer with a stem cell genotype and or phenotype is termed “*degree of stemness*”. Data in diverse cancers such as colorectal and esophageal cancers indicate correlations between the *degree of stemness* and clinical outcomes such as therapy-response, progression-free survival (PFS), and survival (6–8). There are also correlations between the *degree of stemness* with the cancer micro-environment, including immune and stromal cell scores.

The function of stem cells in lymphoma is attracting increasing attention, and increased stemness is related to the formation and progression of lymphoma. It has been hypothesized that an aberrant epigenetic state as the first hit in stem/progenitor cells that retain stem cell-like features or reprogram stemness into mature cells, followed by subsequent genetic aberrations and chromosomal instability eventually lead to lymphoma formation (9). As the same time, Das et al. revealed that cancer stem cells exhibit higher tumorigenic capacity about 5,724-fold compared with non-cancer stem cells in lymphoma (10). Similarly, Chen et al. found that significantly increased proportion of cancer stem cells in R-CHOP-resistant DLBCL cells, whose stemness was regulated by the activated PI3K/AKT1/SOX2 axis (11).

We studied the *degree of stemness* based on RNA expression data from 1,398 subjects with diffuse large B-cell lymphoma (DLBCL). We found correlations with micro-environmental immune and stromal cell scores and clinical outcomes. We identified *CDC7* as a key gene whose expression correlates with the *degree of stemness* and show CDC7-inhibitor reverse growth of human lymphoma cell lines *in vitro* and in a mouse xenograft model.

## Materials and methods

### Data collection and processing

RNA expression data and corresponding clinical data from 1,398 subjects were analyzed from the Gene Expression Omnibus (GEO) database under accession numbers GSE117556 and GSE31312. *Degree of stemness* was calculated using the single-sample gene set enrichment analysis (ssGSEA) and gene set variation analysis (GSVA) packages in R, and a modified version of a 109-gene set derived from Miranda et al. (6).

### Correlation between the *degree of stemness* and immune micro-environment

The gene expression-based de-convolution algorithm (CIBERSORTx, https://cibersortx.stanford.edu/index.php) was used to score the proportion of ten merged immune cell types and 22 detailed immune cells (12). The ESTIMATE package was used to calculate immune and stromal scores defined as proportions of these cells in a sample (13).

### Co-expression network construction and identification of stemness-related modules and key genes

Raw micro-array data from the sequence record GSE117556 were used to construct co-expression networks and screen for key genes. Differentially expressed genes (DEGs) with a false discovery rate (FDR) < 0.025 and |log2FC| > 0.1 between the low- and high-stemness groups in GSE117556 were screened. Weighted correlation network analysis (WGCNA) was conducted on DEGs. A scale-free co-expression network was developed using the WGCNA algorithm (14). A “signed network adjacency” matrix was first constructed from the gene expression data of the GSE117556 cohort of 928 subjects using a soft thresholding power of 7, which was selected to ensure scale-free topology and provide sufficient node connectivity.

The adjacency matrix was then transformed into a “topological overlap matrix (TOM)” to minimize the effects of noise and spurious associations, and the corresponding dissimilarity was calculated as “1 – TOM”. Hierarchical clustering of the dissimilarity was performed using an “average” linkage method to produce a clustering tree of genes in which the branches of the clustered groups of genes were highly interconnected. The gene network modules were identified by cutting the branches off the clustering tree using the “DynamicTreeCut” R package, setting a cut-height value of 0.99, deep split of 2, and minimum module size of 25. Key genes associated with stemness were identified based on the Spearman correlation coefficient between the module and gene expression profiles in the respective modules. DisNor (https://disnor.uniroma2.it/) explored gene-disease interaction networks by exploiting the explosion of data on the identification of disease-associated genes (15). We explored the human protein atlas (https://www.proteinatlas.org) for the CDC7 protein expression in lymph tissue.

### Cell viability and apoptosis assays of *CDC7*-inhibitors

Dequalinium chloride (hereafter dequalinium, a CDC7-inhibitor) and simurosertib (a CDC7-inhibitor) were obtained from Topscience (Shanghai, China) and MedChemExpress (Monmouth Junction, NJ, USA). DLBCL cell lines SU-DHL-2 and SU-DHL-10, verified by short tandem repeats (STR), were cultured in Roswell Park Memorial Institute (RPMI)-1640 medium (Gibco, Grand Island, NY, USA) supplemented with 10% fetal bovine serum (Biochrom AG, Berlin, Germany). Cells were seeded into 96-well plates at a density of 10^5^ cells/well. To determine the cell inhibition rate of the inhibitors, cells were incubated at appropriate inhibitor concentrations for 24, 48, 72, and 96 h; 20 ul of the reagent from the cell counting kit-8 (CCK-8, APExBio, Houston, TX, USA) was added to each well followed by incubation for 2 h at 37°C and absorbance at 450 nm determined using a microplate reader. An Annexin V-fluorescein isothiocyanate (FITC)/propidium iodide (PI) staining kit (FA111-02, Transgen Biotech, Beijing, China) was used to quantify apoptosis induced by exposure of cells to the inhibitors for 48 h. A cell-cycle kit (KGA511, KeyGENE, Nanjing, China) was used to analyze the cell-cycle following exposure to the *CDC7*-inhibitors for 48 h.

### Immune blotting

SU-DHL-2 and SU-DHL-10 cells were treated with dequalinium and simurosertib for 4, 6, and 12 h. Cells were lysed using RIPA (Radio Immunoprecipitation Assay Lysis buffer) and total protein separated by sodium dodecyl sulfate-polyacrylamide gel electrophoresis (SDS-PAGE) and transferred onto a polyvinylidene fluoride (PVDF) membrane (Bio-Rad, Hercules, CA, USA). Primary antibodies were used at a concentration of 0.1 to 0.5 mg/mL, including anti-CDC7 (R23882, ZENBIO, Chengdu, China), anti-mini-chromosome maintenance protein 2 (MCM2, Ser 53; AF8489, Affinity Biosciences, Liyang, China), cleaved caspase-3(9661T, CST, Danvers, USA) and anti-glyceraldehyde 3-phosphate dehydrogenase (GAPDH; ab9484, Abcam, Cambridge, UK). Membranes were probed using primary antibodies at 4°C overnight followed by incubation with anti-rabbit or anti-mouse secondary antibodies for 2 h. Immunoblotted proteins were visualized by chemoluminescence (Bio-Rad).

### Plasmid delivery

293T cells were seeded in a 6-well plate and cultured overnight. Transfection was done when there was approximately 75% confluence (Lipofectamine 3000 kit; Invitrogen, Carlsbad, CA, USA). SU-DHL-10 was infected by incubation with a solution containing the lentivirus collected from the 6-well plate. Stable cell lines over-expressing *CDC7* or the empty plasmid were selected using puromycin 48 h after infection. SU-DHL-10 cells over-expressing *CDC7* or the empty plasmid were seeded into 96-well plates at a density of 5x10^3^ cells/well, and 20 μL CCK-8 was added to each well after 24, 48, 72, and 96 h of incubation followed by incubation for 2 h at 37°C, and absorbance at 450 nm was measured using a micro-plate reader.

### *CDC7*-inhibitor studies

SU-DHL-10 and SU-DHL-10 cells over-expressing *CDC7* or the empty plasmid (1 × 10E+7) were suspended in 50% Matrigel (scorning, NY, USA) and 50% phosphate-buffered saline (PBS, Gibco) and injected subcutaneously into 5–6-week-old male BALB/c nude mice obtained from Gempharmatech-GD (Nanjing, China). Mice with SU-DHL-10-induced tumors with a mean tumor size of 150–300 mmE+3 were randomly divided into control (double distilled water (dd H2O)- or dequalinium-treated cohorts. Dequalinium, 15 mg/kg administered orally, was given every 3 days until day 33 when the tumor size of the control cohort exceeded the ethical limit. Tumor volume (V) was calculated as V = (A × BE+2) × 0.52 (A, long diameter; B, short diameter; mmE+3). The wet weight of the tumor was measured after tumor removal from the mice. The animal studies complied with research guidelines and were approved by the Animal Ethics Committee of Sun Yat-sen University Cancer Center (2021-000096).

### Histologic analysis

Tumors were fixed in 4% paraformaldehyde (BL539A, biosharp, Hefei, China) for 24 h, and embedded in paraffin. Tumor sections (3 μm) were cut, followed by deparaffinization, heat antigen retrieval and endogenous peroxidase blocking of the tumor sections. Subsequently, the tumor sections were blocked with 3% bovine serum albumin in PBS for 30 min and incubated with anti-human Ki-67(ZM-0167, ZSGB-BIO, Beijing, China), BCL-2(ZM-0167, ZSGB-BIO, Beijing, China) and cleaved caspase-3(9661T, CST, Danvers, USA) antibody for overnight at 4°C. Biotinylated goat anti-mouse IgG (PV-6000, ZSGB-BIO, Beijing, China) were then added and incubated for 50 min. Detection was conducted with DAB detection kit according to manufacturer’s instructions. The tumor sections were counterstained with hematoxylin. Slides were scanned on an OLYMPUS Microscope and the whole mount digitalized at 10x magnification. Three regions in each sample were randomly selected and analyzed using Image J.

### Statistical analyses

We used restricted cubic spline (RCS) Cox regression with four knots with the inflection point set as reference testing for linearity and exploring the relationships between the *degree of stemness* with PFS and survival in DLBCL (16). PFS was defined as the interval from diagnosis to progression or death. Survival was defined as the interval from diagnosis to death from any cause. PFS and survival were compared using Kaplan–Meier (KM) plots and the log-rank test. Uni- and multi-variable Cox regressions were done to identify prognostic performance. Distributions of clinical co-variates were analyzed by Wilcoxon or Kruskal–Wallis tests. Pearson correlation analyses combined statistical significance testing was used to evaluate correlations between the *degree of stemness* with proportions of immune and stromal cells. The proportion of lymphoma cells in the sample was tested, and immune and stromal scores were compared with the *degree of stemness* cohorts by the Wilcox test. A nomogram was used to visualize the model of multi-variable Cox regression, which integrated the *degree of stemness* and the International Prognostic Index (IPI) to construct the merged model (data on R-IPI were not contained in the datasets). Performance was assessed for predictive accuracy and calibrated for PFS and survival. The R 3.6.0 software (https://www.R-project.org) was used for all statistical analyses. Statistical significance was set at *P* < 0.05.

## Results

### Data collection and study population

Gene expression and clinical data from 1,398 subjects with DLBCL were downloaded from the GEO database (sequence records GSE117556 and GSE31312). There were 571 males (56%) in the GSE117556 dataset and 271 (58%) in the GSE31312 dataset. Median ages were 64 years (Interquartile Range [IQR], 56-71 years) and 64 years (IQR, 54-74 years). The proportions of GCB, ABC subtypes, and unclassified were 51%, 26%, and 23% in GSE117556 and 48%, 42%, and 9% (*P* = 0.02) in GSE31312, respectively. Proportions of stage I-IV in GSE117556 were 3%, 28%, 31%, and 38%. Stage data were unavailable in the GSE31312 dataset. Distributions of IPI scores are displayed in **Table 1**.

**Table 1:** Distribution of clinical co-variates.

Cohort	GSE117556	GSE31312	*P-*value
**Sample**	928	470	
Male	517 (56%)	271 (58%)	0.23
Age > 65	457(49%)	207 (44%)	0.07
**Sub-types**			0.02
GCB	475 (51%)	227 (48%)	
ABC	244 (26%)	199 (42%)	
NA	209 (23%)	44 (9%)	
**Stage**			NA
Stage I	27 (3%)	NA	
Stage II	259 (28%)	NA	
Stage III	283 (31%)	NA	
Stage IV	355 (38%)	NA	
NA	4 (0%)	NA	
**IPI**			0.80
Low	246 (27%)	169 (36%)	
Low-intermediate	236 (25%)	105 (22%)	
Intermediate-high	281 (30%)	92 (20%)	
High	165 (18%)	58 (12%)	
NA	NA	46 (10%)	

NA, Not available.

### Correlations with the *degree of stemness*

**Figure 1:**
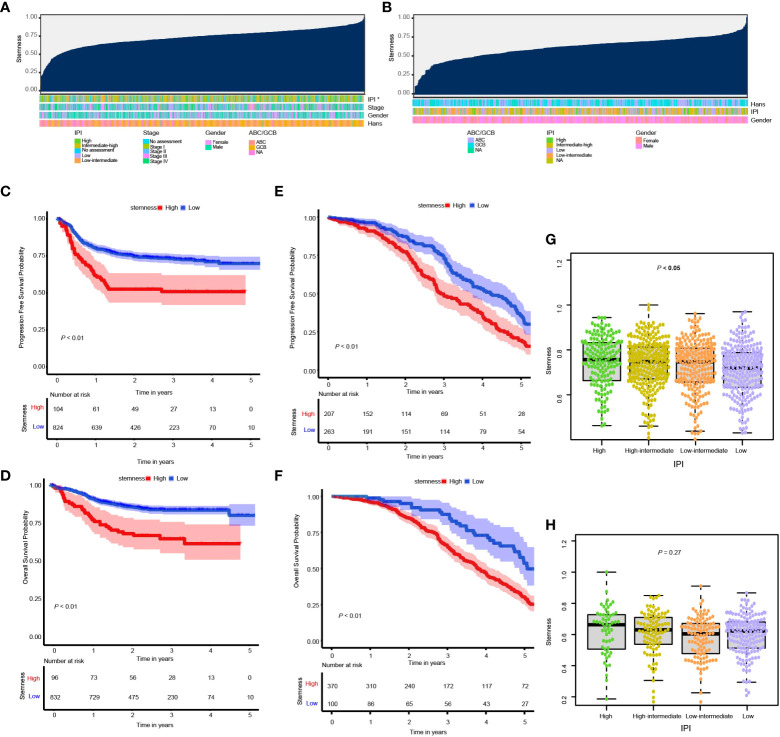
**(A, B)** Overview of the correlation between potential predictive co-variates and the *degree of stemness* in the GSE117556 and GSE31312 datasets. Columns represent samples sorted by the *degree of stemness* from low to high. Rows represent potential predictive co-variates; **(C, D)** Kaplan–Meier (K-M) analyses of PFS and survival in cohorts with low or high *degrees of stemness* in the GSE117556 dataset; **(E, F)** K-M analyses of PFS and survival in cohorts with low or high *degrees of stemness* in the GSE31312 datasets; **(G, H)** Boxplots of the *degree of stemness* in individual subjects stratified by the international prognostic index (IPI) in the GSE117556 and GSE31312 datasets. *P < 0.05.

In the training cohort subjects, there were significant correlations between the *degree of stemness* with IPI cohorts of high, high-intermediate, low-intermediate, and low (**Figures 1A, B, G, H** **)** as well as the double *MYC*/*BCL2* expressor but not with sex, age, stages, *MYC*/*BCL2*/*BCL6* rearrangement or ABC vs GBC type (**Supplementary Figure 1**). No significant correlations were found in the validation cohort.

### *Degree of stemness* and prognosis

RCS was used to visualize the relationship of the *degree of stemness* with PFS and survival in the GSE117556 and GSE31312 datasets. We found the hazard ratio (HR) for death to be relatively flat until a *degree of stemness* of approximately 0.74 and 0.76 above which it rapidly increased and non-linearly correlated (*P-*values for non-linearity < 0.001; **Supplementary Figures 2A-D** **)**. Consequently, we chose these values to divide subjects into low- and high-*degree of stemness* cohorts in the subsequent analyses.

In the GSE117556 dataset, the 3-year PFS of the high and low *degree of stemness* cohorts were 51% (95% Confidence Interval [CI], 42%, 62%) vs 73% (70%, 76%; *P* < 0.001; Hazard Ratio [HR] =1.90 [1.37, 2.64]; *P* < 0.001), and survivals were 65% (55%, 76%) vs 83% (81%, 86%; *P* < 0.001), with a HR of 2.29 [1.53, 3.44]; *P* < 0.001). In the GSE31312 dataset, the 3-year PFS were 50% (43%, 59%) vs 75% (68%, 81%; *P* < 0.001; HR = 1.51 [1.19, 1.90]; *P* = 0.001), and survivals were 66% (60%, 71%) vs 88% (80%, 96%; *P* < 0.001; HR = 2.10 [1.53, 2.88]; *P* < 0.001). The *degree of stemness* was independently correlated with PFS and survival in both dataset in multi-variable Cox regression analyses (**Figures 1C–F**; **Tables 2**, **3**; **Supplementary Tables 1, 2**).

**Table 2:** Multi-variable Cox regression analyses of clinical co-variates, *degree of stemness* and progression-free survival.

	*P-*value	HR (95% CI)
GSE117556 cohort		
Age	< 0.01	0.98 (0.97, 1.00)
IPI (Low intermediate vs. Low)	0.01	1.72 (1.16, 2.56)
IPI (Intermediate-high vs. Low)	< 0.001	2.02 (1.38, 2.96)
IPI (High vs. Low)	< 0.001	3.33 (2.16, 5.11)
*Degree of stemness* (High vs. Low)	< 0.001	1.90 (1.37, 2.64)
GSE31312 cohort		
Age	0.01	1.37 (1.08, 1.76)
IPI (Low intermediate vs. Low)	0.31	1.17 (0.86, 1.58)
IPI (Intermediate-high vs. Low)	0.52	1.12 (0.79, 1.59)
IPI (High vs. Low)	0.66	0.90 (0.56, 1.44)
*Degree of stemness* (High vs. Low)	0.001	1.51 (1.19, 1.90)

HR, Hazard Ratio; CI, confidence interval.

**Table 3:** Multivariable Cox regression analyses of clinical features, *degree of stemness* and survival.

	*P-*value	HR (95% CI)
GSE117556 cohort		
ABC vs. GCB	0.01	1.61 (1.11, 2.34)
IPI (Low intermediate vs. Low)	0.07	1.72 (0.96, 3.08)
IPI (Intermediate-high vs. Low)	0.001	2.48 (1.44, 4.25)
IPI (High vs. Low)	< 0.001	4.21 (2.35, 7.53)
*Degree of stemness* (High vs. Low)	< 0.001	2.29 (1.53, 3.44)
GSE31312 cohort		
IPI (Low intermediate vs. Low)	0.26	1.19 (0.88, 1.59)
IPI (Intermediate-high vs. Low)	0.76	0.95 (0.67, 1.33)
IPI (High vs. Low)	0.60	1.13 (0.71, 1.80)
*Degree of stemness* (High vs. Low)	< 0.001	2.10 (1.53, 2.88)

HR, Hazard Ratio; CI, confidence interval.

The IPI score and *degree of stemness* were integrated into a merged model to predict the 5-year survival probability in the training and validation cohorts (**Supplementary Figure 3A**). The merged model had a higher AUROC compared with the IPI, i.e., 0.70 (0.63, 0.73) vs 0.68 (0.63, 0.73; *P* < 0.01; **Supplementary Figure 3B**) and 0.51 (0.46, 0.56) vs 0.54 (0.49, 0.59; *P* < 0.01; **Supplementary Figure 3C**) in the training and validation cohorts, respectively. Calibration plots of the model showed good agreement between estimated and survival probabilities (**Supplement Figures 3D, 3E**).

### Correlations between the *degree of stemness* and immune and stromal cell scores

**Figure 2:**
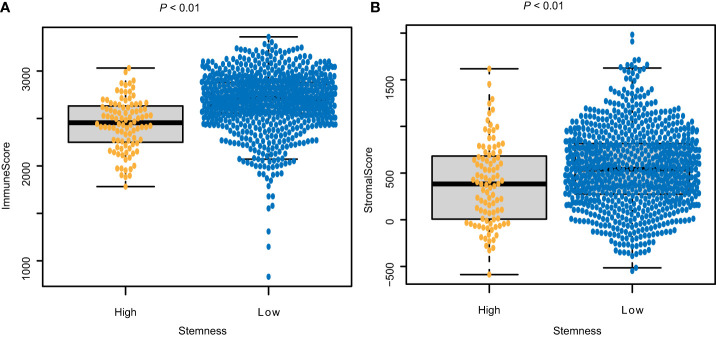
Correlations between the *degree of stemness* and **(A)** Immune score; and **(B)** stromal score.

**Figure 3:**
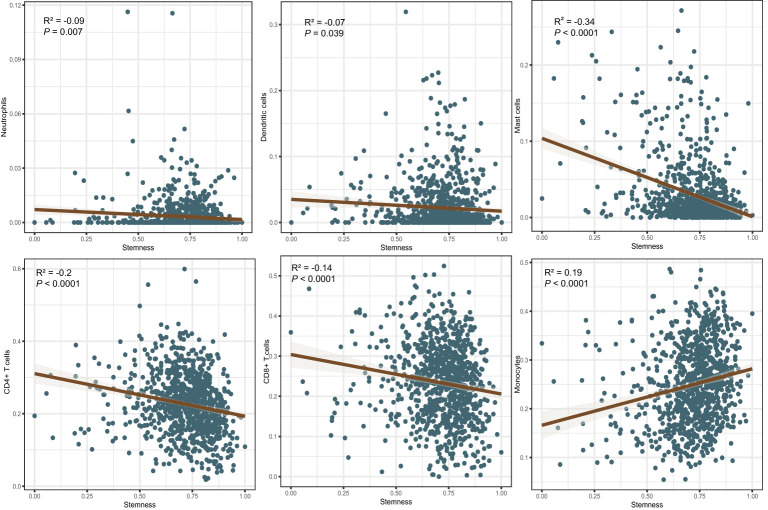
Correlations between the *degree of stemness* and immune cell type.

The immune score of the high *degree of stemness* cohort was significantly lower than that of the low *degree of stemness* cohort (2,437 ± 270 vs 2,686 ± 291; P < 0.05; **Figure 2A** **)**. Immune cell types were also correlated with the *degree of stemness*. The *degree of stemness* was negatively correlated with the fraction of CD4-positive T-cells (coefficient of determination (R^2^) = -0.2; *P* < 0.001), CD8-positive T-cells (R^2^ = -0.14; *P* < 0.001), neutrophils (R^2^ = -0.09, *P* = 0.01), mast cells (R^2^ = -0.34; *P* < 0.001), activated mast cells (R^2^ = -0.32, *P* < 0.001), eosinophils (R^2^ = -0.09; *P* = 0.01), dendritic cells (R^2^ = -0.07, *P* = 0.04) and activated dendritic cells (R^2^ = -0.08; *P* = 0.02). In contrast, there were positive correlations with the fraction of monocytes (R^2^ = 0.19; *P* < 0.001), CD4-posoitive memory-activated T-cells (R^2^ = 0.16; *P* < 0.001), and M0 macrophages (R^2^ = 0.15, *P* < 0.001), M1 macrophages (R^2^ = 0.17, *P* < 0.001), and activated NK-cells (R^2^ = 0.06, *P* < 0.05; **Figure 3**; **Table 4**).

**Table 4:** Correlations between *degree of stemness* with immune cell types.

Immune cell	R2	*P-*value
CD4+ T-cells	-0.20	<0.001
CD8+ T-cells	-0.14	<0.001
CD4+ T-cells naïve	-0.30	<0.001
Tregs	-0.10	< 0.01
Gamma delta T-cells	-0.22	<0.001
Follicular helper T-cells	-0.08	0.02
Resting memory CD4 T-cells	-0.08	0.01
Activated memory CD4 T-cells	0.16	<0.001
NK cells	0.05	0.14
Resting NK cells	-0.04	0.18
Activated NK cells	0.06	0.05
Monocytes	0.19	<0.001
Macrophages M0	0.15	<0.001
Macrophages M1	0.17	<0.001
Macrophages M2	0.00	0.97
Dendritic cells	-0.07	0.04
Resting dendritic cells	0.04	0.18
Activated dendritic cells	-0.08	0.02
Neutrophils	-0.09	0.01
Mast cells	-0.34	<0.001
Resting mast cells	-0.03	0.34
Activated mast cells	-0.32	<0.001
Eosinophils	-0.09	0.01

R2, Correlation coefficient.

The stromal score of the high *degree of stemness* cohort was significantly lower compared with the low *degree of stemness* cohort (388 ± 440 vs. 551 ± 415; *P* < 0.05, **Figures 2B**).

### Construction of weighted co-expression network and identification of key modules

**Figure 4:**
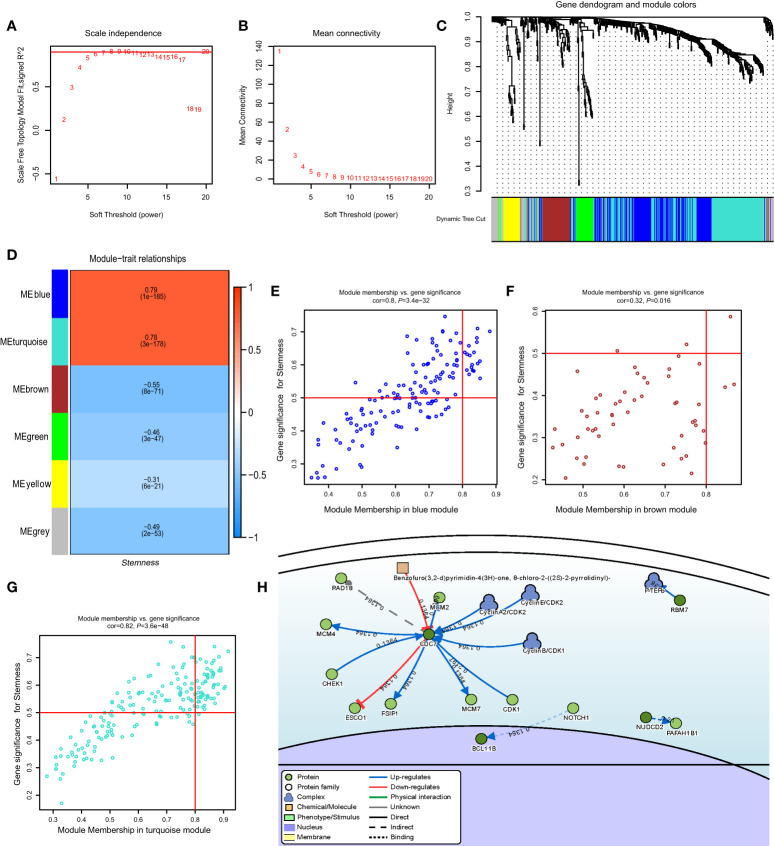
**(A)** Relationship of soft-threshold (power) with scale-free topology; **(B)** Relationship of soft-threshold (power) with mean connectivity; **(C)** WGCNA analysis of DEG. Different colors correspond to related modules; **(D)** Correlation coefficient between modules and the *degree of stemness*. *P*-values are listed in modules; **(E–G)** Scatter plot analysis of different modules in MEblue, MEbrown, and MEturquoise modules; **(H)** Gene-disease interaction network analyses of key genes in DisNor. WGCNA, Weighted Gene Co-expression Network Analysis; DEG, Differentially Expressed Genes.

In the GSE117556 dataset, we identified 491 differentially expressed genes (DEGs) between the high and low *degree of stemness* cohorts (**Supplementary Figures 4A, B**). Next, we performed a WGCNA of these DEGs. A threshold power of β = 7 was selected to construct a scale-free network, and six modules were further identified. The turquoise and blue modules had significant positive correlation indices with the *degree of stemness*, with correlation indices of 0.79 (*P* = 1 × 10^−185^) and 0.78 (*P* = 3 × 10^−178^). The brown, green, yellow, and grey modules had negative correlation indices of -0.55 (*P* = 8 × 10^−71^), -0.46 (*P* = 3 × 10^−47^), -0.31 (*P* = 6 × 10^−21^), and -0.49 (*P* = 2 × 10^−53^; **Figures 4A–G**). In uni-variate analyses, the expression of 74 genes correlated between the module and *degree of stemness* > 0.5 and between genes and module > 0.8, indicating that 16 genes were correlated with survival (**Supplementary Figure 4C**). In multi-variate Cox regression analyses, the expression of 4 genes showed that *CDC7* (HR = 1.24 [1.03, 1.50]; *P* = 0.02), *SLC16A1* (HR = 1.37 [1.12, 1.67]; *P* < 0.01), *CCDC41* (HR =1.30 [1.07, 1.57]; *P* = 0.01), and *LOC440145* (HR =1.40 [1.09, 1.80]; *P* = 0.01) were independently correlated with survival (**Table 5** **;** **Supplementary Table 3**).

**Table 5:** Multi-variable Cox regression analyses of key genes in GSE117556.

	*P-*value	HR (95%CI)
ABC vs. GCB	0.02	1.54 (1.06, 2.22)
IPI (Low-intermediate vs. Low)	0.07	1.73 (0.97, 3.09)
IPI (Intermediate-high vs. Low)	0.001	2.58 (1.51, 4.43)
IPI (High vs. Low)	<0.001	4.42 (2.47, 7.89)
*CDC7*	0.02	1.24 (1.03, 1.50)
*SLC16A1*	< 0.01	1.37 (1.12, 1.67)
*CCDC41*	0.01	1.30 (1.07, 1.57)
*LOC440145*	0.01	1.40 (1.09, 1.80)

HR, Hazard ratio; CI, confidence interval.

### Key gene identification and validation

**Figure 5:**
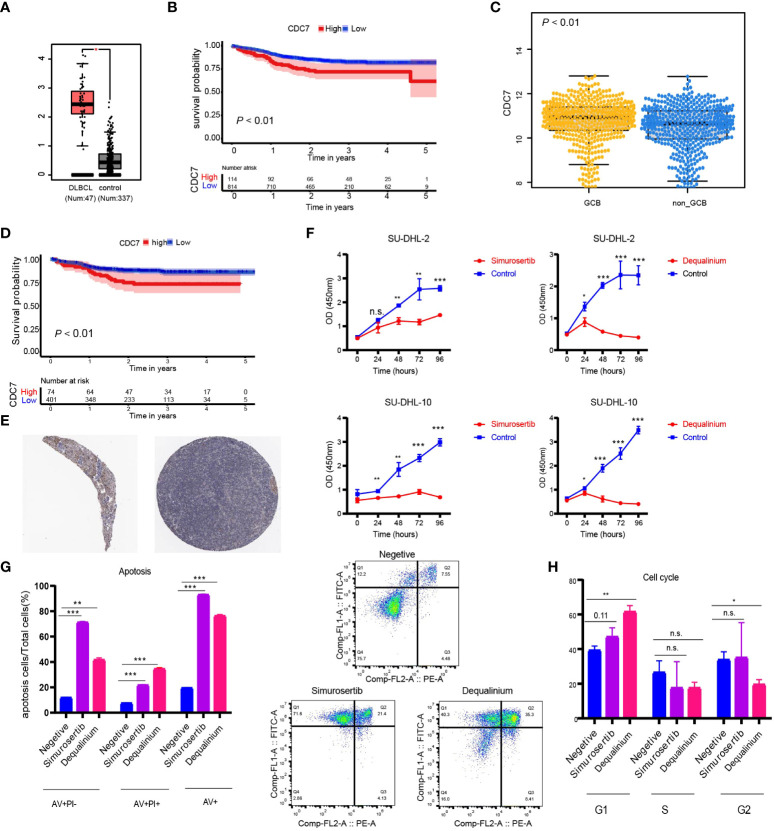
**(A)** Expression level of *CDC7* in normal and lymphoma samples from the TCGA database; **(B)** Kaplan-Meier (K-M) curves of the expression of *CDC7*; **(C)** Boxplots of the expression of *CDC7* in samples stratified by ABC/GBC subtype; **(D)** Kaplan-Meier (K-M) curves of the expression of *CDC7* for GCB cohort; **(E)** Immune histochemistry of *CDC7* in normal lymph node; **(F)** Viability of SU-DHL-2 and SU-DHL-10 cells treated with dequalinium and simurosertib by IC_50_; **(G)** Apoptosis ratio analysis of SU-DHL-10 cells treated with dequalinium and simurosertib for 48 h; **(H)** cell cycle analysis of SU-DHL-10 cells treated with dequalinium and simurosertib for 48 h. Data are means ± standard deviation (SD) of triplicate experiments. Dequalinium and simurosertib compared with the control using unpaired t-tests (*P < 0.05, **P < 0.001, and ***P < 0.0001). n.s., no significants.

Using DisNor (https://disnor.uniroma2.it/), we identified *CDC7* as a hub gene correlated with the *degree of stemness*, which was druggable (**Figure 4H**). In the cancer genome atlas (TCGA), *CDC7* expression was lower in samples from normal persons compared with that in samples from persons with DLBCL, based on gene expression profiling and interactive analyses (*P* < 0.05; **Figure 5A**) (17). The expression of CDC7 was divided into high and low groups based optimal cut-off value and we found that high *CDC7* expression was associated with poor survival (*P* < 0.01, **Figure 5B**). We also found that *CDC7* expression in the GCB subtype was higher compared with the ABC subtype (*P* < 0.01, **Figure 5C**). The *CDC7* expression level was independently correlated with survival (HR = 1.24 [1.03, 1.50]; *P* = 0.02; **Table 5**) but not with PFS *(P* = 0.18).

Validating protein expression levels of *CDC7* in normal lymph nodes, the human protein atlas (www.proteinatlas.org) showed higher expression in GBC vs ABC, results consistent with those previously reported (**Figure 5E**) (18–24). In survival analyses, high *CDC7* expression in subjects with the GCB type was worse compared with subjects with low *CDC7* expression (*P* < 0.01; **Figure 5D**).

### CDC7 inhibitors suppress lymphoma growth *in vitro*

In the CCK-8 assay, half-maximal inhibitory concentration (IC_50_) values of dequalinium in the SU-DHL-2 and SU-DHL-10 cells were 3.119 µmol and 7.347 µmol. Corresponding IC_50_ values for simurosertib were 8.718 µmol and 13.636 µmol (**Figure 5F**). The percentages of apoptotic cells were 20%, 77 ± 1%, and 93% in control, dequalinium-, and simurosertib-treated SU-DHL-10 cells. Inhibiting CDC7 significantly promoted the apoptosis of treated vs untreated cells (*P* < 0.01, **Figure 5G**). G_1_-phase was prolonged in SU-DHL-10 cells after dequalinium treatment (62 ± 3% vs 39 ± 2%; *P* < 0.01) and simurosertib (47 ± 4% vs 39 ± 2%; *P* = 0.11, **Figure 5H**).

**Figure 6:**
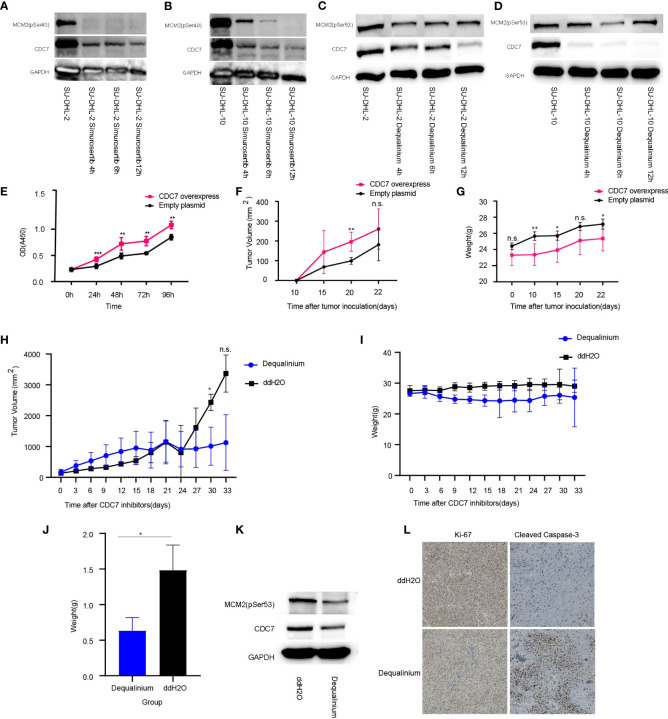
CDC7 over-expression promotes lymphoma growth, which is reversed by dequalinium. **(A, B)** Effect of simurosertib-treated SU-DHL-2 and SU-DHL-10 cells after incubation for 4, 6, and 12 h *in vitro*. **(C, D)** Effect of dequalinium-treated SU-DHL-2 and SU-DHL-10 cells after incubation for 4, 6, and 12 h *in vitro*. **(E)** Viability of CDC7 over-expression using the cell counting kit-8 (CCK-8); **(F)** Lymphoma volume of BALB/c mice with *CDC7*-over-expressing and empty plasmid-transfected SU-DHL-10 cells; **(G)** Mice weight of BALB/c mice with *CDC7*-over-expressing and empty plasmid-transfected SU-DHL-10 cells; **(H)** Lymphoma volume of BALB/c mice implanted with SU-DHL-10 cells given dequalinium or placebo as described; **(I)** Mice weight of BALB/c mice implanted with SU-DHL-10 cells given dequalinium or placebo; **(J)** Weight of tumors removed from BALB/c mice implanted with SU-DHL-10 cells receiving dequalinium or placebo; **(K)** Effect of dequalinium-treated lymphoma from mice; **(L)** Immunohistochemistry was conducted for lymphoma from mice. Tumors were stained for human Ki-67 and human cleaved caspase-3. Data are means ± standard deviation (SD), *P < 0.05, **P < 0.001 and ***P < 0.0001, n.s., no significants.

Dequalinium and simurosertib inhibited CDC7 activity in a time-dependent manner in SU-DHL-2 and SU-DHL-10 cells to different extents, as determined by the MCM2 phosphorylation intensity at Ser53 and Ser40 (**Figures 6A–D**). Specifically, the protein expression levels of CDC7 and MCM2 (pSer40) were lower in simurosertib-treated SU-DHL-2 and SU-DHL-10 cells after 12 h than they were after 4 h. Furthermore, the protein expression of CDC7 and MCM2 (pSer53) was lower in dequalinium -treated SU-DHL-2 and SU-DHL-10 cells at 6–12 h than it was at 4 h.

### *CDC7* expression increases lymphoma growth *in vitro* and *in vivo*

In the CCK-8 assay, *CDC7* over-expression increased proliferation (*P* < 0.001, **Figure 6E**). Nude mice subcutaneously injected with SU-DHL-10 cells over-expressing CDC7 developed significantly larger tumors and lower body weight compared with mice injected with SU-DHL-10 cells with empty plasmid (mean volume at 20 days: 196 ± 44 mmE+3 vs 99 ± 12 mmE+3, *P* < 0.001; mean weight at 22 days: 25 ± 1 g vs 27 ± 1 g, *P* < 0.05, **Figures 6F, G** **)**. Gene Set Enrichment Analysis (GSEA) was used to explore molecular mechanism of CDC7 in GCB and non-GCB patients. The results show that there were significant differences in the activities of regulation of autophagy pathway and protein export pathway in higher expression CDC7 group compared with lower expression CDC7 group in GCB patients. As the same time, there was no difference pathway between the higher and lower expression CDC7 groups in non-GCB patients (**Supplementary Figure 5A**).

### Dequalinium inhibits lymphoma growth *in vivo*

Mice were implanted with SU-DHL-10 cells and received dequalinium, 15 mg/kg administered orally every 3 days, beginning on day 1 until day 30. Controls received dd-H_2_O orally using the same schedule. 30-day lymphoma volumes were 1,012 ± 504 mmE+3 vs 2,432 ± 215 mmE+3 (*P* < 0.05). There was no weight loss in mice receiving dequalinium compared with the controls (**Figures 6H–J**; **Supplementary Figure 5B**). The antitumor effects of dequalinium in lymphoma was verified which were collected from mice at the end of the study. Western blot showed that the protein expression of CDC7 and MCM2(pSer53) were down-regulated in tumor sample treated by dequalinium (**Figure 6K**). Ki-67 and cleaved caspase-3 expression in lymphoma was examined by immunohistochemistry in both dequalinium and control group. The results show that Ki-67 expression was lower in the dequalinium group relative to dd-H_2_O. As the same time, cleaved caspase-3 was higher in the dequalinium group relative to dd-H_2_O (**Figure 6L**). The results showed that dequalinium could target CDC7 to inhibit lymphoma proliferation and promote lymphoma apoptosis *in vivo* and consistent with *in vitro*.

## Discussion

Our data indicate correlations between *degree of stemness*, immune and stromal scores and clinical outcomes in samples from persons with DLBCL. Several related studies have shown that stemness indices were negatively correlated with prognosis in diverse cancers such as leukemia, esophageal cancer, and medulloblastoma, consistent with our results in DLBCL (3, 6, 8, 25, 26). We also show that *CDC7* expression was strongly correlated with the *degree of stemness*, and that the CDC7-inhibitors dequalinium and simurosertib inhibited growth of human DLBCL cell lines *in vitro* and in a mouse xenograft model.

We have confirmed the *degree of stemness* in 1,398 subjects and identified two clear prognostic groups. We also integrated the *degree of stemness* with the IPI, which produced a more accurate prediction model. On the one hand, the *degree of stemness* could add tumor biological features for IPI. On the other hand, combination of the IPI with the *degree of stemness* could result in a better treatment decision. For instance, drugs such as dequalinium and simurosertib targeting stem-related genes were given to the higher stemness group. In addition, recent studies report that enhancing the IPI with positron emission tomography (PET) data (Metabolic Prognostic Index (IMPI)) increased prediction accuracy (27, 28). Due to the lack of PET data on our subjects, we could not compare our combined model with the IMPI. As a consequence, continued efforts are needed to construct excellent prognostic scoring models to better risk-stratify DLBCL patients and select those for novel therapies.

Miranda et al. speculated that tumor stemness could lead to tumor heterogeneity by protecting tumor clones from being recognized by immune cells (6). Cytostatic factors secreted by cancer stem cells or related ligands on the surface of cancer stem cells promote tumor immune escape in several cancers involved in AML and melanoma, which increases the tumorigenic growth of tumors (29, 30). We found that the *degree of stemness* was negatively correlated with immune activation of cells in the lymphoma micro-environment, including dendritic and mast cells. This phenomenon may indicate that tumor stemness inhibits activated immune cells and drives tumor escape. Previous data indicate that macrophages regulate the activities of cancer stem cells (31, 32). M1 macrophages contribute to immunity to cancer *via* pro-inflammatory cytokines, reactive nitrogen and oxygen intermediates, and a complex network of NF-κB, Stat-1/4, and IRF1/5 (33).

CDC7 is a serine–threonine kinase required to initiate DNA replication and acts together with the cyclin-dependent kinase CDK2. Human CDC7 phosphorylates MCM2, a component of the DNA replicative helicase is required for genome duplication (34). In tumors cells, the appropriate response of CDC7 to replication stress plays an important role in tumor transformation by triggering a series of replication activation and ATR–CHK1 checkpoint responses (35–37). In terms of prognosis, a high percentage of CDC7-positive cells has been reported in several cancers, including DLBCL, and correlates with a poor prognosis (22, 38). Hou et al. reported that *CDC7* silencing combined with rituximab increased apoptosis of DLBCL cell lines (21). This research found that the activities of regulation of autophagy pathway and protein export pathway in higher expression CDC7 group in GCB patients. Wang et al. reported that autophagic activate autophagic flux in ATPase ATP6V1B2 mutation follicular lymphoma, as the same time, Primary human FL B cells carrying mutant ATP6V1B2 are sensitive to inhibition of autophagic flux (39). As the same time, Gayle et al. provide evidence that apilimod induces significant cytotoxicity by inhibition of the autophagy flux in preclinical models of B-cell non-Hodgkin lymphoma (NHL) (40). As a consequence, toward autophagy-targeted therapy is worthy of further verification in lymphoma.

Dequalinium, a broad-spectrum antibiotic and non-ATP-competitive CDC7 kinase inhibitor, inhibits CDC7 kinase activity, S-phase progression, and accumulation in G2/M phase (41). Simurosertib was developed by Iwai et al., which is a novel CDC7-selective inhibitor and caused mitotic abnormalities by centrosome and chromatin dysregulation during cancer cell replication (42). In terms of efficacy, dequalinium and simurosertib exhibited significant antiproliferative activity in large-scale cell panel data and preclinical animal models. Concordant with this, our results show that simurosertib and dequalinium can inhibit cell proliferation, induce apoptosis, and arrest the cell cycle *in vitro* and *in vivo* for DLBCL. Specifically, dequalinium is an FDA-approved broad-spectrum antibiotic, and the discovery of antitumor effect on DLBCL. This not only reduces the costs for drug development but also enables complete toxicological clinical trials and shortens the time required from clinical trial to clinical application.

Our study has some limitations. First, although we report correlations between *degree of stemness* immune and stromal scores and clinical outcomes, the precise mechanism(s) underlying these correlations are unknown and cannot be assumed to be causal relations. Second, we lacked data on some important predictive co-variates in both datasets, including mutation topography, Ki-67, R-IPI, and PET scan data. Third, there is well-known heterogeneity in cell composition of lymphoma samples, including needle biopsies from the same and different lymphoma sites. Finally, the biological function and molecular mechanism of CDC7 in DLBCL need to be clarified further.

In conclusion, we report correlations between *degree of stemness*, immune and stromal scores and PFS and survival in samples from persons with DLBCL. These data will improve prediction of therapy outcomes in DLBCL and suggest potential new therapies.

## Data availability statement

The raw data supporting the conclusions of this article will be made available by the authors, without undue reservation.

## Ethics statement

The animal study was reviewed and approved by Sun Yat-sen University Cancer center.

## Author contributions

Conceptualization, RG and YL; methodology, FH, HL, and LL; validation, HL and LL; formal analysis, YS and LL; investigation, FH, LL, and HL; resources, HL; data curation, SC and LL; writing—original draft preparation, FH, and HL; writing—review and editing, RG and YL; visualization, FH; supervision, RG and YL; project administration, RG and YL; funding acquisition, YL. All authors contributed to the article and approved the submitted version.

## Funding

Funded, in part, by the National Natural Science Foundation of China (NSFC Grant No. 81873428) and the Program for Guangdong Introducing Innovative and Entrepreneurial Teams (2017ZT07S096).

## Acknowledgments

RG acknowledges support from the National Institute of Health Research (NIHR) Biomedical Research Centre funding scheme.

## Conflict of interest 

RG is a consultant to NexImmune Inc. and Ananexa Pharma Ascentage Pharm Group, Antengene Biotech LLC, Medical Director, FFF Enterprises Inc.; partner, AZAC Inc.; Board of Directors, Russian Foundation for Cancer Research Support; and Scientific Advisory Board: StemRad Ltd.

The remaining authors declare that the research was conducted in the absence of any commercial or financial relationships that could be construed as a potential conflict of interest.

## Publisher’s note

All claims expressed in this article are solely those of the authors and do not necessarily represent those of their affiliated organizations, or those of the publisher, the editors and the reviewers. Any product that may be evaluated in this article, or claim that may be made by its manufacturer, is not guaranteed or endorsed by the publisher.

## Supplementary material

Supplementary Figure 1
**(A–G)** Boxplots of the *degree of stemness* in samples stratified by sex, age, stage, ABC/GBC, MYC/BCL2/BCL6 rearrangement, expressor of MYC/BCL2 by immunohistochemistry (IHC), and expressor of MYC/BCL2 by RNA-seq subtype in GSE117556. **(H–J)** Boxplots of the *degree of stemness* in samples stratified by age, sex, and ABC/GBC subtype in GSE31312. IHC, Immune histochemistry.

Supplementary Figure 2
**(A)** Restricted cubic spline analyses of the association between PFS and the *degree of stemness* in GSE117556. **(B)** Restricted cubic spline analyses of the association between survival and the *degree of stemness* in GSE117556. **(C)** Restricted cubic spline analyses of the association between PFS and the *degree of stemness* in GSE31312. **(D)** Restricted cubic spline analyses of the association between survival and the *degree of stemness* in GSE31312; HR, hazard ratio.

Supplementary Figure 3 Merged risk score construction. **(A)** Merged model for survival prediction was constructed by a nomogram based on the *degree of stemness* and IPI in the GSE117556 dataset; **(B)** Comparison of the AUROC of the merged and single prediction models in the GSE117556 dataset; **(C)** Comparison of the AUROC of the merged and single prediction models in the GSE31312 dataset. **(D)** Calibration curve of the merged model indicating agreement between predicted and observed survival in the GSE117556 datasett; **(E)** Calibration curve of the merged model indicating agreement between predicted and observed survival in the GSE31312 dataset.

Supplementary Figure 4
**(A)** Heatmap of differentially expressed genes (DEGs) between high and low *degree of stemness* cohorts; **(B)** Volcano plot of DEGs; **(C)** Uni-variate analyses of key genes. DEGs, differentially expressed genes.

Supplementary Figure 5
**(A)** Gene set enrichment analysis (GSEA) results between the higher expression CDC7 group and lower expression CDC7 group in GCB patients. **(B)** General shape of tumor of SU-DHL-10 cells receiving dequalinium or placebo.
