# Supplementary figures and images for: Degree of stemness predicts micro-environmental response and clinical outcomes of diffuse large B-cell lymphoma and identifies a potential targeted therapy
Source: Front Immunol. 2022 Nov 8;13:1012242. doi: 10.3389/fimmu.2022.1012242 (PMC9678919; doi:10.3389/fimmu.2022.1012242)

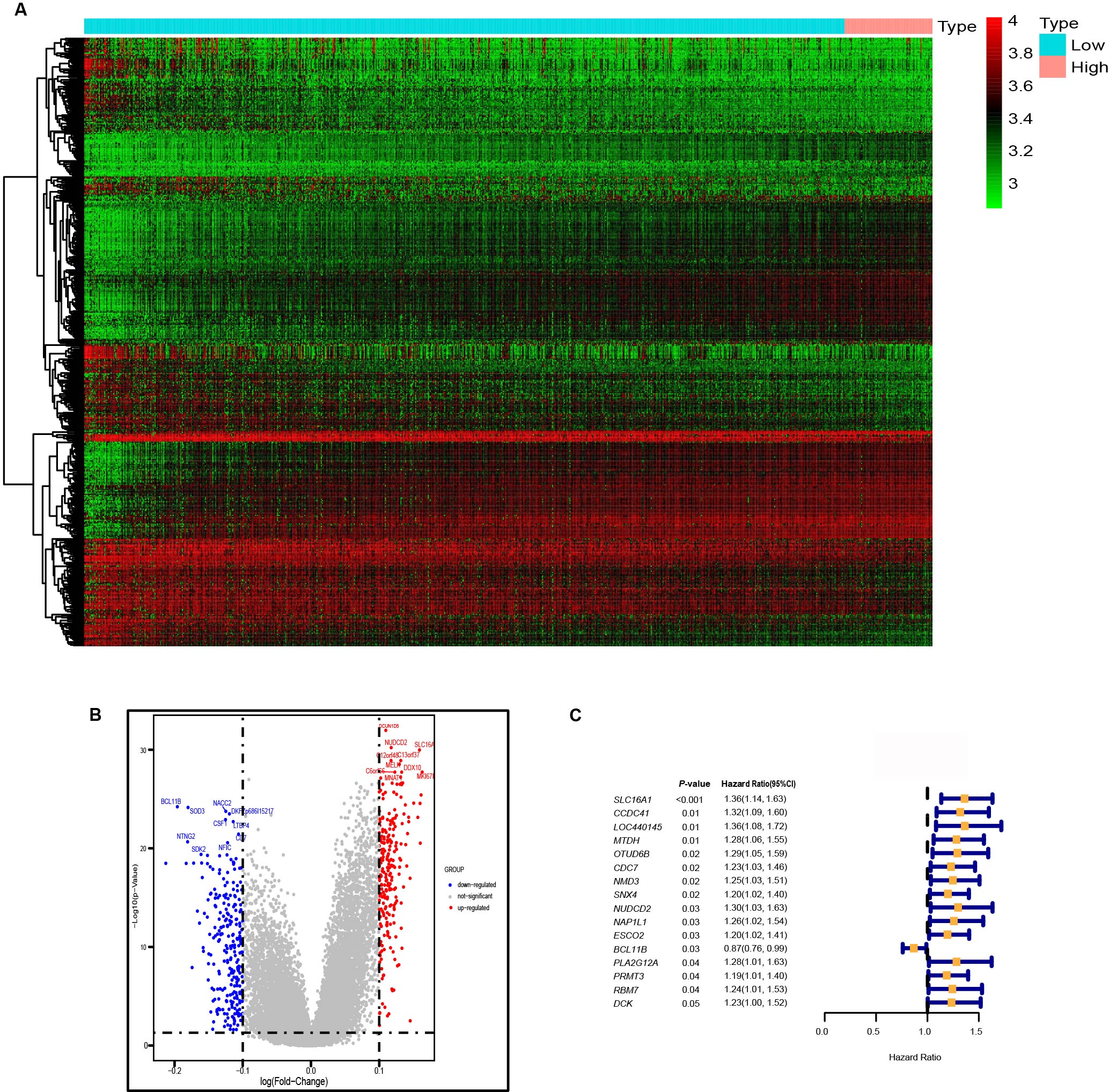

Supplement: Supplementary Figure 4 — (A) Heatmap of differentially expressed genes (DEGs) between high and low degree of stemness cohorts; (B) Volcano plot of DEGs; (C) Uni-variate analyses of key genes. DEGs, differentially expressed genes. [file Image_4.jpg]

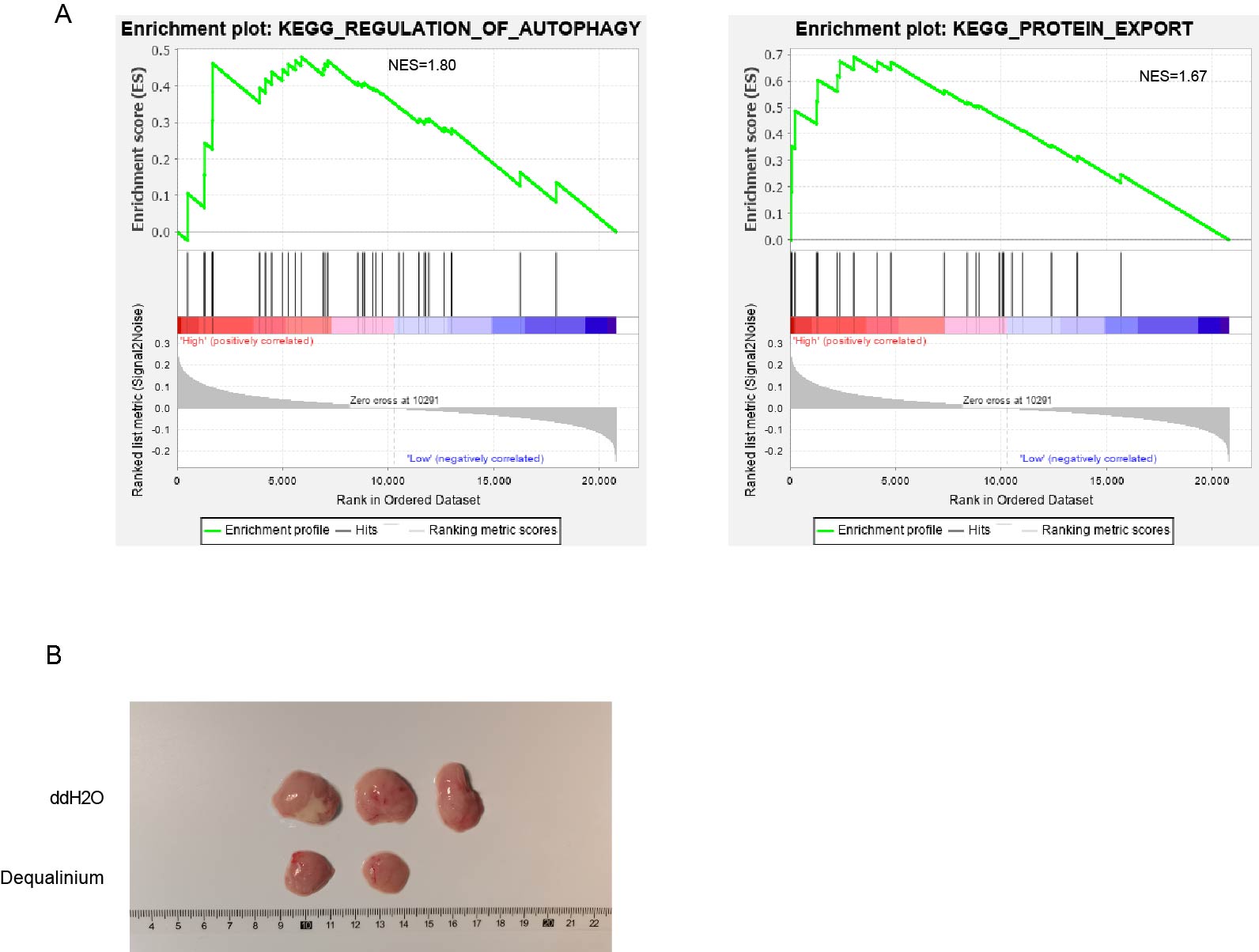

Supplement: Supplementary Figure 5 — (A) Gene set enrichment analysis (GSEA) results between the higher expression CDC7 group and lower expression CDC7 group in GCB patients. (B) General shape of tumor of SU-DHL-10 cells receiving dequalinium or placebo. [file Image_5.jpg]
