# Supplementary material for: Degree of stemness predicts micro-environmental response and clinical outcomes of diffuse large B-cell lymphoma and identifies a potential targeted therapy
Source: Front Immunol. 2022 Nov 8;13:1012242. doi: 10.3389/fimmu.2022.1012242 (PMC9678919; doi:10.3389/fimmu.2022.1012242)
Supplement: Supplementary file 6 [file Table_1.docx]

**Supplement table 1.** Univariable Cox regression analyses of clinical co-variates, *degree of stemness* and progression-free survival.

|  | **Uni-variable** | |
| --- | --- | --- |
|  | *P-*value | HR (95%CI) |
| GSE117556 cohort |  |  |
| Age | 0.69 | 1.00 (1.00-1.01) |
| ABC vs. GCB | 0.13 | 1.25 (0.94-1.66) |
| Female vs. Male | 0.63 | 1.06 (0.83-1.36) |
| Stage (Stage II vs*.* Stage I) | 0.58 | 0.80 (0.36-1.76) |
| Stage (Stage III vs*.* Stage I) | 0.82 | 0.91 (0.42-2.00) |
| Stage (Stage IV vs*.* Stage I) | 0.22 | 1.62 (0.76-3.45) |
| IPI (Low intermediate vs*.* Low) | **0.02** | 1.56 (1.06-2.31) |
| IPI (Intermediate-high vs*.* Low) | **0.001** | 1.82 (1.26-2.64) |
| IPI (High vs*.* Low) | **< 0.001** | 2.93 (1.94-4.43) |
| *Degree of stemness* (High vs. Low) | **< 0.001** | 2.13 (1.54-2.94) |
| GSE31312 cohort |  |  |
| Age | **0.01** | 1.41 (1.11-1.80) |
| Gender (Female vs*.* Male) | 0.69 | 1.05 (0.83-1.33) |
| IPI (Low intermediate vs. Low) | 0.19 | 1.22 (0.91-1.65) |
| IPI (Intermediate-high vs. Low) | 0.26 | 1.22 (0.86-1.73) |
| IPI (High vs*.* Low) | 0.98 | 1.01 (0.63-1.60) |
| ABC vs*.* GCB | 0.97 | 1.01 (0.67-1.51) |
| *Degree of stemness* (High vs. Low) | **< 0.001** | 1.53 (1.21-1.93) |

Note: Bold indicates P < 0.05; HR, Hazard Ratio; CI, confidence interval
