# Supplementary material for: Degree of stemness predicts micro-environmental response and clinical outcomes of diffuse large B-cell lymphoma and identifies a potential targeted therapy
Source: Front Immunol. 2022 Nov 8;13:1012242. doi: 10.3389/fimmu.2022.1012242 (PMC9678919; doi:10.3389/fimmu.2022.1012242)
Supplement: Supplementary file 7 [file Table_2.docx]

**Supplement table 2.** Univariable Cox regression analyses of clinical features, degree of stemness and survival.

|  | **Univariable** | |
| --- | --- | --- |
|  | *P* | HR (95%CI) |
| GSE117556 cohort |  |  |
| Age | **0.01** | 1.02 (1.01-1.04) |
| ABC vs. GCB | 0.05 | 1.44 (1.00-2.08) |
| Gender (Female vs. Male) | 0.69 | 1.03 (0.88-1.21) |
| Stage (Stage II vs. Stage I) | 0.08 | 0.45 (0.19-1.09) |
| Stage (Stage III vs. Stage I) | 0.31 | 0.64 (0.17-1.51) |
| Stage (Stage IV vs. Stage I) | 0.91 | 1.05 (0.46-2.41) |
| IPI (Low intermediate vs*.* Low) | 0.05 | 1.80 (1.01-3.21) |
| IPI (Intermediate-high vs. Low) | **< 0.001** | 2.65 (1.54-4.53) |
| IPI (High vs*.* Low) | **< 0.001** | 4.44 (2.49-7.92) |
| *Degree of stemness* (High vs. Low) | **< 0.001** | 2.50 (1.69-3.69) |
| GSE31312 cohort |  |  |
| Age | 0.08 | 1.24 (0.98-1.58) |
| Gender (Female vs. Male) | 0.67 | 1.05 (0.84-1.33) |
| IPI (Low intermediate vs*.* Low) | 0.37 | 1.15 (0.85-1.54) |
| IPI (Intermediate-high vs. Low) | 0.50 | 0.89 (0.63-1.25) |
| IPI (High vs. Low) | 0.73 | 1.08 (0.68-1.72) |
| ABC vs. GCB | 0.36 | 1.12 (0.88-1.43) |
| *Degree of stemness* (High vs. Low) | **0.01** | 1.53 (1.13-2.08) |

Note: Bold indicates P < 0.05; HR, Hazard Ratio; CI, confidence interval
