# Supplementary material for: Degree of stemness predicts micro-environmental response and clinical outcomes of diffuse large B-cell lymphoma and identifies a potential targeted therapy
Source: Front Immunol. 2022 Nov 8;13:1012242. doi: 10.3389/fimmu.2022.1012242 (PMC9678919; doi:10.3389/fimmu.2022.1012242)
Supplement: Supplementary file 8 [file Table_3.docx]

**Supplement table 3.** Univariable Cox regression analyses of key genes in GSE117556

|  | **Univariable** | |
| --- | --- | --- |
|  | *P* | HR (95%CI) |
| AGE | **0.01** | 1.02 (1.01-1.04) |
| ABC vs. GCB | 0.05 | 1.44 (1.00-2.08) |
| Gender (Female vs. Male) | 0.69 | 1.03 (0.88-1.21) |
| Stage (Stage II vs. Stage I) | 0.08 | 0.45 (0.19-1.09) |
| Stage (Stage III vs. Stage I) | 0.31 | 0.64 (0.17-1.51) |
| Stage (Stage IV vs. Stage I) | 0.91 | 1.05 (0.46-2.41) |
| IPI (Low intermediate vs. Low) | 0.05 | 1.80 (1.01-3.21) |
| IPI (Intermediate-high vs. Low) | **< 0.001** | 2.65 (1.54-4.53) |
| IPI (High vs. Low) | **< 0.001** | 4.44 (2.49-7.92) |
| *CDC7* | **0.02** | 1.23 (1.03-1.46) |
| *SLC16A1* | **< 0.001** | 1.36 (1.14−1.63) |
| *CCDC41* | **0.01** | 1.32 (1.09−1.60) |
| *LOC440145* | **0.01** | 1.36 (1.08−1.72) |

Note: Bold indicates P < 0.05; HR, Hazard Ratio; CI, confidence interval
